# Ethnobotanical Analysis and Regional Comparison of Ethnoveterinary Practices in Southern Fars, Iran

**DOI:** 10.1155/sci5/1812536

**Published:** 2025-11-30

**Authors:** Saeideh Ghafouri, Roja Safaeian, Gholamabbas Ghanbarian, Thea Lautenschläger, Ehsan Ghafouri

**Affiliations:** ^1^Department of Natural Resources and Environmental Engineering, School of Agriculture, Shiraz University, Shiraz, Fars, Iran; ^2^Institute for Botany, University of TU Dresden, Dresden, Germany

**Keywords:** ethnobotanical analysis, ethnoveterinary medicine, Fars, Iran, regional comparison

## Abstract

This article analyzes the traditional ethnoveterinary knowledge of medicinal plants among the Bikheii, Korosh, and Achomi tribes in Fars, Iran. Ethnoveterinary data collected from 200 informants in 27 local communities were analyzed using the ethnobotanyR package. The analysis identified 31 plant species from 21 families used in ethnoveterinary practices. The most commonly used plant families were Rosaceae and Fabaceae. *Ferula assa-foetida* and *Astragalus fasciculifolius* had the highest use reports (URs) and were noted as having particular importance in the daily lives of tribal people in the south of Fars in Iran. A key focus of this study is a regional comparison with other documented ethnoveterinary practices, primarily within Iran, to identify conserved knowledge and novel findings. This study contributes to the conservation and sustainable use of traditional ethnoveterinary knowledge, which has previously been limited to herders and aged community members. The findings also provide a basis for future phytochemical and pharmacological studies to validate the efficacy of these medicinal plants for veterinary purposes.

## 1. Introduction

Throughout history, plants have served as a vital source of food and shelter for humans and animals, while also offering natural remedies to manage and alleviate diseases [1]. Ethnoveterinary medicine draws on traditional knowledge and practices passed down through generations to care for animals. This includes using readily available plants and herbs with known medicinal properties to treat livestock diseases and maintain animal health. This approach is sustainable and eco-friendly, making it especially valuable for rural communities and small-scale farmers who may have limited access to modern veterinary medicine [2].

The literature on plants used in ethnoveterinary medicine is increasing including [1–10] around the world and [11–14] in Iran.

Ethnoveterinary medicine plays a crucial role in animal health care in many developing countries, yet it remains poorly documented. Further research and integration efforts are needed in these regions. Iran, with its diverse climate (11 different types), abundant sunshine (300 days annually), and significant temperature variations (up to 50°C between coldest and warmest locations), offers unique ecological conditions. This favorable environment supports a rich flora, encompassing over 7500 plant species, many of which possess medicinal properties [13].

Iran's legacy as one of the world's earliest civilizations includes a rich and continuous history of traditional medicine, with origins tracing back to the Babylonian–Assyrian era. This medical heritage flourished during the Islamic Golden Age, when prominent Iranian scholars and physicians such as Avicenna, Rhazes, and Jorjani made foundational contributions to the medical sciences. The framework they helped establish, known as Iranian traditional medicine, is a holistic system principally founded on the Unani tradition of ancient Persia.

A central tenet of this enduring system is its reliance on medicinal plants. Although Iranian traditional medicine incorporates other natural resources, herbal remedies are its primary component, a fact confirmed by modern ethnobotanical surveys. This deep-rooted tradition of using therapeutic plants is reflected in Iran's status as one of Asia's leading consumers of medicinal herbs, with over 130 officially registered plant-based medicines.

This rich medical history is further amplified by the nation's unique convergence of high biodiversity and significant ethnic diversity. Home to numerous communities including Persians, Turks, Arabs, Turkmen, Lurs, Kurds, and Baloch, Iran's multicultural landscape provides a robust foundation for a deep and varied body of ethnobotanical knowledge, making it a crucial area for ethnopharmacological research [15].

The southern Fars Province of Iran is home to diverse ethnic groups with deep-rooted traditional knowledge. This study focuses on three prominent tribes in the region: the Korosh, the Bikheii, and the Achomi. The Korosh are a branch of the Baloch tribe who migrated from the Sistan and Baluchestan provinces to Fars in the past, maintaining a nomadic lifestyle until recently settling in the area. According to local accounts, some of the Bikheii people descend from the Lurs of Bushehr Province who migrated to Fars. The Achomi people, an ancient Persian ethnic group, have inhabited the southern regions of Iran for generations, tracing their roots to the old Persian language and culture. This article presents findings from the first comprehensive ethnobotanical study conducted among these specific tribes in this region. It examines the use of medicinal plants for animal treatments, utilizing data from a larger ethnobotanical study [16] to facilitate a regional comparison with other Iranian ethnoveterinary research.

## 2. Materials and Methods

**Figure 1:**
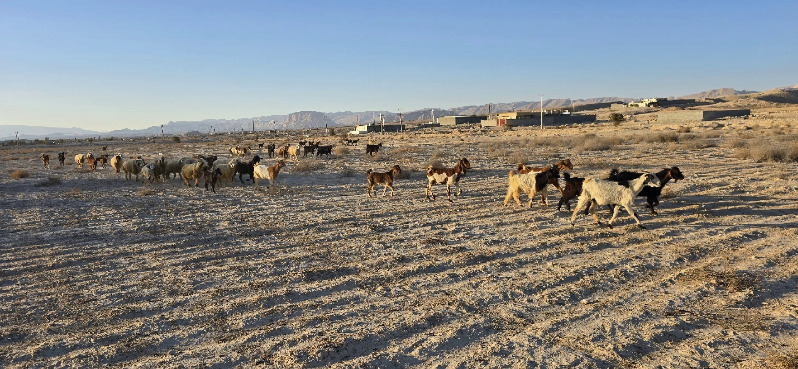
A view of the study area and its livestock.

The ethnoveterinary data were extracted from a larger ethnobotanical dataset collected between 2020 and 2022 from 200 informants in 27 local communities. Semi-structured interviews were conducted using snowball sampling to identify knowledgeable individuals from the Bikheii, Korosh, and Achomi tribes [16]. A view of the study area and its livestock is shown in Figure 1.

### 2.1. Plant Collection and Identification

Plant specimens were collected during field visits conducted between 2020 and 2022, often accompanied by local informants. The collected specimens were pressed, dried, and mounted on herbarium sheets for preservation and identification.

The scientific identification of the plant specimens was a multistep process. It was based on the morphological characteristics of the plants (fruits, flowers, leaves, stems, etc.) and cross-referenced with authoritative botanical literature, including “Identification of Medicinal and Aromatic Plants of Iran” [17], “Dictionary of Iranian Plant Names” [18], and the “Flora of Lamerd” [19]. To confirm the identifications, the online databases of the [20], [21], and [22] were utilized. All authenticated voucher specimens were deposited in the herbarium of the Fars Agricultural and Natural Resources Research and Education Center.

The identifications were verified by a taxonomist at the Fars Agricultural and Natural Resources Research and Education Center, and the voucher specimen codes are listed in Table 1.

### 2.2. Regional Comparison

The present study focuses on the ethnobotanical analysis and interpretation of this ethnoveterinary data subset, with a particular emphasis on regional comparison with other Iranian studies. For the regional comparison, a comprehensive literature review of ethnoveterinary studies published in national and international journals was conducted. The documented uses of the 31 plant species identified in our study were then systematically compared against these published findings, as summarized in Table 1.

### 2.3. Data Analysis

To quantify the collected ethnoveterinary data, several well-established ethnobotanical indices were calculated using the ethnobotanyR package in R [37]. These indices help to objectively assess the cultural and medicinal importance of different plant species. The following indices were used.

Use reports (URs) and frequency of citation (FC) were used to count the number of uses reported for a species and the number of informants who cited it, respectively [37, 38]. Relative FC (RFC) was calculated to reflect the local importance of each species, based on the number of informants who mentioned it [39]. Use value (UV) was used to measure the relative importance of a plant based on its number of reported uses [38]. Informant consensus factor (ICF) was calculated to measure the level of agreement among informants regarding the use of plants for specific ailment categories. A higher ICF value (closer to 1) indicates strong consensus [40, 41]. Fidelity level (FL) was used to determine the percentage of informants who reported the use of a certain plant for a specific purpose, helping to identify the most preferred species for treating a particular ailment [42, 43]. Choice value (CV) was used to further identify the most preferred plant species for treating a specific disease category [44]. Cultural importance index (CI) was calculated to assess not just the prevalence of a plant's use but also the diversity of its applications [39, 45].

## 3. Results

**Figure 2:**
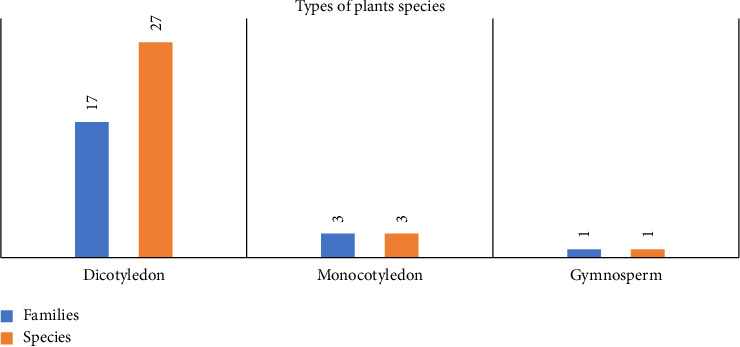
Types of plant species.

In total, 31 plant species belonging to 21 families were identified. Of these, three belong to monocots, 27 belong to dicots, and one family belongs to gymnosperms (Figure 2). The plant species identified in this study, along with their botanical classification, local names, habit, habitat, and conservation status, are mentioned in detail in our first publication of the region [16]. Here, we present a summary of key characteristics relevant to the ethnoveterinary context.

**Figure 3:**
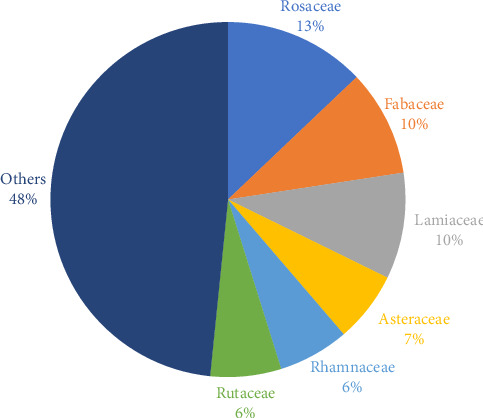
Most representative families.

Rosaceae, Fabaceae, and Lamiaceae were the most commonly used plant families in the study area (Figure 3).

### 3.1. Plants for Veterinary Medicine

In this region, 31 plant species are used for the treatment of livestock diseases (Table 1), a subset of the data presented in Ghafouri et al. [16]. The detailed information in Table 1 is crucial for the comparative analysis of regional ethnoveterinary practices developed in this study. These plants are primarily used for gastrointestinal issues. The most commonly used plants include *Ferula assa-foetida* Boiss. and *Astragalus fasciculifolius* Boiss., and following them is *Periploca graeca* L. The reported uses of the identified plants were compared with those documented in 30 ethnoveterinary studies conducted in Iran and other parts of the world, as detailed in Table 1.

Only one combination drug was mentioned for the treatment of animal diseases, which is used to prevent abortion, increase weight and milk production, and eliminate parasites and treat epistaxis in animals (Table 2).

### 3.2. Mode of Administration

**Figure 4:**
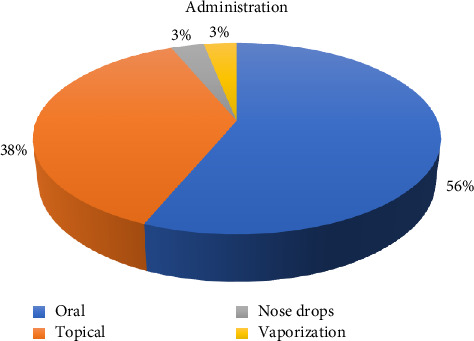
Routes of administration in veterinary.

The methods of administration of animal medicines include oral (56%), topical (38%), nasal drops (3%), and vaporization (3%) (Figure 4).

### 3.3. Quantitative Analysis of the Reported Plants

The UV for veterinary medicines in the region ranged from 0.005 to 0.44. The most important medicinal plants for veterinary use in terms of UV include the following: *Ferula assa-foetida* Boiss., *Astragalus fasciculifolius* Boiss, *Amygdalus lycioides* Spach*, Capparis spinosa* L., *Otostegia persica* (Burm.) Boiss., *Tecomella undulata* (Sm.) Seem., *Periploca graeca* L., *Ziziphus nummularia* (Burm.f.) Wight. & Arn., and *Ziziphus spina-christi* (L.) Desf (Table 3).

The most important medicinal plants based on RFC are as follows: *Ferula assa-foetida* Boiss., *Astragalus fasciculifolius* Boiss., *Periploca* graeca L., *Amygdalus lycioides* Spach, *Capparis spinosa* L., *Otostegia persica* (Burm.) Boiss., *Ziziphus nummularia* (Burm.f.) Wight. & Arn., *Ziziphus spina-christi* (L.) Desf., *Haplophyllum laristanicum* C.C.Towns., and *Tecomella undulata* (Sm.) Seem (Table 3).

The ICF value for animal diseases is shown in Table 4.

By calculating the CV for each disease, the best plant for treating the respective disease according to the local people's perspective can be determined. Plants with the highest CV for treating animal diseases are listed in Table 5.

The FC ranged from 1 to 58. *Ferula assa-foetida* Boiss., commonly known as “Anghoozeh,” is a widely used plant in the treatment of animal diseases, with a citation frequency of 58. *Astragalus fasciculifolius* Boiss. was mentioned 14 times. *Periploca graeca* L. had a citation frequency of 6, whereas *Amygdalus lycioides* Spach, *Capparis spinosa* L., *Otostegia persica* (Burm.) Boiss., *Ziziphus nummularia* (Burm.f.) Wight. & Arn., and *Ziziphus spina-christi* (L.) Desf. were each mentioned five times (Table 3).

The plants with the highest UR are *Ferula assa-foetida* Boiss., *Astragalus fasciculifolius* Boiss., and *Amygdalus lycioides* Spach (Table 3).

The FL ranges from 1.72% to 100%. There are 30 cases with a FL of 100%, as shown in Table 6. For example, Achillea eriophora DC. is used for digestive problems, Otostegia persica (Burm.) Boiss. for fractures, Sesamum indicum L. for muscular pain, Eruca sativa Miller for scabies disease in camels, Calotropis procera (Aiton) W.T.Aiton for infected wounds, Ephedra pachyclada Boiss. for nosebleeding, digestive problems, muscular pain, and increasing lactation, Phoenix dactylifera L. for grass tetany, and Tecomella undulata (Sm.) Seem. for animal bite caused by lizard.

### 3.4. Comparative Analysis of Reported Uses

**Figure 5:**
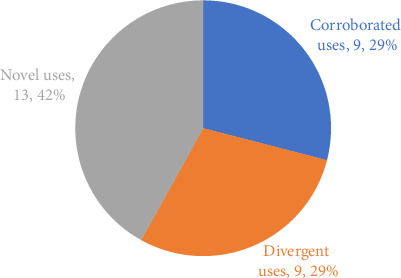
Comparative analysis of ethnoveterinary plant uses in southern Fars against published literature.

A comparative analysis of the 31 documented ethnoveterinary plant uses was conducted against published national and international literature. The results of this comparison, which categorizes each use based on its relationship to existing reports, are presented in Figure 5.

Of the 31 plant applications, a total of 13 uses (42%) were identified as novel uses. This category represents therapeutic applications that were not found in the reviewed literature, highlighting previously undocumented knowledge. An additional nine uses (29%) were classified as divergent uses, where a plant is used for a different therapeutic purpose in our study region compared to reports from other areas. The remaining nine uses (29%) were categorized as corroborated uses, where our findings are similar to and validated by existing published data. Notably, the largest portion of the documented practices represents novel applications, underscoring the unique ethnoveterinary knowledge held by the communities in southern Fars.

## 4. Discussion

### 4.1. Knowledge Conservation and Novelty in Ethnoveterinary Practices

These findings corroborate previous research highlighting the importance of traditional knowledge in animal health care in Iran and other regions. The study identified both conserved knowledge and novel applications of medicinal plants. This is reflected in our quantitative analysis (Figure 5), which found that the majority of documented practices were either novel (42%) or divergent (29%) from the established literature, highlighting the unique knowledge base of this region.

For example, the use of *Ferula assa-foetida* for intestinal worm elimination and increasing milk production aligns with its reported use in other studies (Abbasnia et al.). In contrast, the application of *Tecomella undulata* for animal bites caused by lizards appears to be a novel finding, specific to the studied communities. Furthermore, significant variations were observed in the use of some plants, for example, *Capparis spinosa*, which the local tribes use for fractures, eliminating worms and external wound infections and uterine wounds, whereas other studies report its use as joint pain, respiratory problems, ulcer, dysentery, sore eyes, and toothache [13, 24, 28, 30]. These variations likely reflect the dynamic nature of traditional knowledge, influenced by local ecological conditions, cultural practices, and intergenerational transmission. The cultural significance of certain plants, such as the use of date syrup (*Phoenix dactylifera*) for grass tetany, underscores the interconnectedness of ethnoveterinary practices with local customs and beliefs.

### 4.2. The “One Medicine” Concept in Traditional Practices

All the plants used in the treatment of animal diseases in this study are widely used in human medicine. However, there are significant differences between human and veterinary medicines. In human treatments, individuals tend to select plant parts more carefully, separating flowers and leaves from stems. However, in traditional veterinary medicine, underground parts, whole plants, resins, and barks are important. Leaves and stems are renewable sources, and their collection does not lead to the death of the entire plant [7]. These mostly indigenous plants are utilized for the treatment of various diseases, primarily minor ailments. It is possible that the recognized and used medications for human health can also be used in the treatment of animals. Additionally, it is also possible that medications that were used by animals for self-medication have later been used for human health care. The specific definitions of diseases, causes, and treatment methods are often applied to both animals and humans in each culture. As a result, remedies that treat humans often also treat animals using similar techniques and substances and vice versa. Among these substances, plants are the most common treatments used, and it has been observed in various parts of the world that similar species can be used for both veterinary and human medical purposes using similar methods [35].

The people of the region believe that in the past, animals did not get sick as much as they do now due to their nomadic lifestyle, and there were fewer problems. The diseases of the animals in the region are mostly seasonal and occasionally epidemic. Livestock, especially during seasonal transitions, are sensitive to changes in vegetation cover and consequently to forage [25]. The most common problems in this area are gastrointestinal issues, especially the treatment of gastrointestinal parasites, as well as fractures and muscular pain.

### 4.3. Limitations of Traditional Dosages

A notable challenge in documenting traditional ethnoveterinary knowledge is the lack of standardized, precise dosages. As observed during our fieldwork, and discussed in detail in our previous work [16], dosages are typically not based on scientific measurements (e.g., mg/kg). Instead, informants rely on qualitative and imprecise units, such as “a handful,” “a small cup,” or “enough to cover the wound.” This lack of standardization is an inherent characteristic of folk medicine systems, where amounts are often adjusted based on the animal's apparent size, age, and the severity of the ailment. Although this qualitative approach is a limitation from a modern pharmacological perspective, it reflects the empirical and personalized nature of traditional healing practices. Therefore, although we have accurately reported the “Mode of Preparation and Administration” as described by the informants, the quantitative amounts could not be reported as they do not exist within this traditional knowledge system.

### 4.4. Cross-Cultural Comparison of Ethnoveterinary Practices

#### 4.4.1. Commonality of Treated Ailments

The prevalence of gastrointestinal issues as the most commonly treated ailment category in our study is a finding that resonates strongly with ethnoveterinary practices across various regions of Iran. This suggests a shared set of health challenges in traditional livestock management. For instance, studies conducted in Golestan Province, the Shahrbabak region of Kerman, and Ilam Province also report digestive disorders, including diarrhea and rumen impaction, as primary concerns for livestock health [23, 31, 46].

Alongside digestive problems, parasitic infections (both internal and external) and the treatment of wounds and skin diseases emerge as other dominant themes in Iranian ethnoveterinary medicine. Research from the Tehran and Azerbaijan provinces highlights a significant focus on treating parasites and skin conditions, whereas work in other parts of Kerman emphasizes musculoskeletal issues and infected wounds ([12, 13, 28]). This cross-regional consistency suggests that ailments related to digestion, parasites, and skin represent a core set of universal health challenges faced by pastoralist communities in Iran, likely stemming from common factors such as forage variability, environmental exposure, and herd management practices.

#### 4.4.2. Comparison of Dominant Plant Families

The dominance of the Rosaceae, Fabaceae, and Lamiaceae families in the ethnoveterinary practices of southern Fars aligns in part with findings from other Iranian regions, while also highlighting distinct regional preferences. The prominence of Fabaceae, for instance, is a shared feature with the ethnoveterinary practices of Azerbaijan, where it is a codominant family, and the Shahrbabak region of Kerman, where it is also frequently used ([12, 31]). Lamiaceae and Rosaceae are other important medicinal families noted in Shahrbabak city of Kerman [31].

However, a notable divergence is the high prevalence of the Asteraceae and Apiaceae in other parts of the country. Asteraceae was found to be the dominant family in studies from Tehran and Kerman, whereas Apiaceae was the most utilized family in Khorasan and Shahrbabak [11, 13, 28, 31]. It suggests that although a core group of medicinally powerful plant families—including Fabaceae, Lamiaceae, Asteraceae, and Apiaceae—forms the backbone of Iranian ethnoveterinary medicine, the specific family most relied upon can differ significantly based on the local flora and the specific traditional knowledge of each community.

#### 4.4.3. Comparison of Administration Methods

The methods of remedy administration documented in our study show a clear preference for oral (56%) and topical (38%) routes. This finding is in strong agreement with practices reported from the Shahrbabak region of Kerman, where oral administration was also the most common method, followed by topical application [31]. The widespread preference for these two routes is logical and practical within a traditional livestock management context. Oral administration is the most direct method for treating internal conditions, such as the prevalent gastrointestinal and parasitic diseases, whereas topical applications in the form of poultices or washes are essential for managing external issues such as wounds, skin infections, and musculoskeletal injuries. This suggests that the primary reliance on oral and topical routes is a highly conserved feature of ethnoveterinary knowledge across different regions of Iran.

## 5. Conclusion

This study successfully documents the rich ethnoveterinary practices of the Bikheii, Achomi, and Korosh tribes in southern Fars, Iran, a traditional knowledge system that is currently declining due to modern innovations. Documenting these 31 medicinal plant uses is therefore crucial for the conservation of this biocultural diversity and for providing a basis for sustainable use. Our findings contribute to a broader understanding of multipurpose plant applications and highlight the need for future research. To build upon this work, priority should be given to investigating the efficacy and safety of these traditional treatments through pharmacological studies, as well as further exploring the cultural and ecological factors that drive the variations in ethnoveterinary practices across different regions.

## Figures and Tables

**Table 1:** List of the medicinal plants used by tribal communities in Larestan, Gerash, and Lamerd (arranged alphabetically according to family).

Botanical name and voucher number	Usage	Plant part	Mode of preparation and administration	Reported usage
Arecaceae				
*Phoenix dactylifera* L./3649	Treatment of grass tetany in livestock	Fruit	During the winter season, a cup of date syrup is given to livestock for the treatment of grass tetany	Poisoning treatment [23], endo- and ectoparasitic [24], for eye diseases, including conjunctivitis. To treat wounds together with wheat [25]
**Asclepiadaceae**				
*Calotropis procera* (Aiton) W.T.Aiton/3324	Infected wounds	Resin	Applying the sap of the plant on a wound that has developed worms helps eliminate the worms	Mange [26], musculoskeletal disorders and dermatological disorders [27]
**Asteraceae**				
*Achillea eriophora* DC./2008	Diarrhea	Aerial parts	*A. eriophora* and *Zataria multiflora* are given to livestock for diarrhea	Bloating [28]
*Matricaria aurea* (Loefl.) Sch.Bip./15063	Reduction in pain and swelling in the postpartum animal's udder and wound healing	Aerial parts	Using the plant steam	_
**Apiaceae**				
*Ferula assa-foetida* Boiss./16371	Treatment for cough, parasites, and muscle stiffness, or when a nursing animal's milk is discontinued (locally known as “Shirvarak”)	Root	The root of the plant is chopped into small pieces and added to the animal's drinking water. The plant roots are left in the drinking water for 10–20 days	Intestinal worm elimination and increasing milk production (Abbasnia et al.)
**Apocynaceae**				
*Periploca graeca* L./7823	Animal bite livestock fracture	Aerial parts	The plant is crushed and boiled, and the thickened poultice is applied to the wound of an animal bitten by a wolf. It is also used for bruises and fractures in livestock	_
**Berberidaceae**				
*Berberis* sp.	Muscle pain	Root	Decoction	_
Bignoniaceae				
*Tecomella undulata* (Sm.) Seem./22400	Animal bite caused by lizard	Bark/resin	The bark of this plant has a sticky sap, which is applied to the affected area when a lizard bites a nursing goat	_
**Brassicaceae**				
*Eruca sativa* Miller/9776	Scabies disease in camels	Seed	Topical application of the oil	Tick infestation [29]
**Capparidaceae**				
*Capparis spinosa* L./1393	Animal fractures Eliminating worms and external wound infections in animals and uterine wounds	Aerial parts Aerial parts	They pound the plant bush and wrap it around the broken leg. They pound the plant, boil it, and pour a few drops on the surface wound of an infected animal where worms have formed, or when a goat gives birth and its uterus is wounded, they pour a few drops inside the goat's uterus	Joint pain killer (horses) [13,28], respiratory problems [24], ulcer, dysentery, sore eyes, toothache [30]
**Fabaceae**				
*Astragalus fasciculifolius* Boiss./1736	Fractures Weaning livestock	Bark/root Resin	The crushed roots of the plant are used for fractures in animals The resin of the plant is applied to a goat's udder for weaning purposes	_
*Glycyrrhiza glabra* L./15485	Fractures	Root/aerial part	Decoction of the plant's root is used for fractures and bruises The leaves and stems of the plant are pounded and heated and then applied for bruises	Diarrhea [31], relieving and reducing pain and swelling in dislocations and bruises, treating gastritis in horses [28], gastrointestinal diseases, otitis (horses) [13], reducing pain and swelling in strains and bruise, stomach swelling in horse [12], cough, bronchitis, toothache [30], enteritis, arthritis [32], endo- and ectoparasitic [24]
Fabaceae				
*Trigonella foenum-graecum* L./10267	Severe diarrhea, cough, and muscle cramps	Seed	The seeds are soaked and either consumed as an extract or used raw for livestock	Diarrhea, mastitis [31], endo- and ectoparasitic [24], wound, colic, paralysis, jaundice [30]. It is used to reduce diseases and increase immunity. For gallbladder disorders and poisoning and to increase lactation [33]. Cure prolapse of the uterus [34]
**Lamiaceae**				
*Otostegia persica (*Burm.f.) Boiss./14093	Fractures	Stem	They crush the peeled stems to use for treating animal fractures	_
*Teucrium polium* L. (C)/14428	Animal bites	Aerial parts	They crush the aerial parts of the plant and apply it to the body of an animal that has been bitten by a wolf, along with warm ashes of tandoor (traditional clay oven)	Treatment of gastrointestinal diseases (cattle and sheep) [13,28,31], constipation, cough, asthma [30], used against vomiting and abdominal pain and wounds [35]
*Zataria multiflora* Boiss./16168	Diarrhea	Leaves	Eating fresh or dry leaves	_
**Moraceae**				
*Ficus johannis* Boiss.	Joint pain Fracture	Fruit Leaves	They combine warm milk and dried figs, and they give it to the animal 2–3 times Decocted leaves are used for setting broken bones	_
**Pedaliaceae**				
*Sesamum indicum* L.	Muscle stiffness and pain	Seed	They pour tahini oil into the nostrils for 2 days, and on the third day, they pour a little thick tahini into the animal's mouth	Diarrhea [29]
Poaceae				
*Triticum aestivum* L.	Diarrhea	Seed	A little flour is poured into water and given to the animal	Mastitis [23], enhancement of lactation [31], bloating, infection, indigestion, anemia, rickets, scabies [12]
**Rhamnaceae**				
*Ziziphus nummularia* (Burm.f.) Wight. & Arn./4776	Poisoning	Root	This decoction is used	Preventing hair loss, mild skin disorders [28]
*Ziziphus spina-christi* (L.) Desf./3431	Diarrhea and strengthening the intestinal villi	Leaves	The plant's leaves are soaked, and their extract is used	
**Rosaceae**				
*Crataegus aronia* (L.) Steud./14186	Urinary retention	Fruit	Consumption of the plant's distillate	_
*Prunus amygdalus* Batsch	Fractures	Fruit	Using almond oil	_
*Prunus eburnea* (Spach) Aitch./8054	Joint pain, infections, and intestinal worms Boosting the overall health and increasing milk production	Whole plant Root	Decoction They boil the plant's roots and give one cup to livestock	Leishmaniasis, *Oestrus ovis* larvae (sheep) ([23]; [12,13])
*Prunus scoparia* (Spach) C.K. Schneid./14059	Fractures	Fruit	Using the fruit peel for treating broken bones	_
**Rubiaceae**				
*Coffea arabica* L.	Diarrhea	Seed	Decoction	Wound, cough, sore eyes, jaundice [30], for wounds and urinary problems and against sunburn [25]
**Rutaceae**				
*Haplophyllum laristanicum* C.C.Towns./25000	Diarrhea in livestock during winter Muscle cramps	Aerial parts Aerial parts	They crush and boil the plant and give 1 cc of the concentrated extract to animals for treating severe diarrhea The decoction of the plant is used for relieving muscle cramps	_
Rutaceae				
*Citrus × aurantiifolia* (Christm.) Swingle	Fever	Fruit	The consumption of dried lime (also known as Omani lime) is cooked for fever in animals	_
**Theaceae**				
*Camellia sinensis* (L.) Kuntze	Grass tetany in livestock	Leaves	They give them a cup of very dark tea	Stomach ache [7,33], endo- and ectoparasitic [24], flatulence, ulcer, constipation, flu [30], fever [4]
**Zingiberaceae**				
*Curcuma longa* L.	Fractures, infected wound	Rhizome	They warm up oil and turmeric and apply it to the bruises. It is also used in combination with other plants for treating infected wounds	Mastitis, wound treatment, joint pain [4], jaundice, enteritis, swellings [30], caprine arthritis encephalitis palliative, proud flesh [36], infectious wound/pyometra [29]

**Table 2:** A complex remedy used by tribal communities in Larestan, Gerash, and Lamerd.

Persian name	Main ingredients	Plant part	Way of preparation and administration	Number of ingredients	Medicinal use
Tengrez	*Prunus eburnea* (Spach) Aitch.	Aerial part/root	Tengrez, Anghuzeh, and Ephedra are decocted together and poured into the animal's throat in the amount of a glass for up to 3 days	3	Preventing miscarriage, weight gain, increasing milk production, treating parasitic infections, and nosebleed
Anghuzeh	*Ferula assa-foetida* Boiss.	Aerial part			
Ephedra	*Ephedra pachyclada* Boiss./5827	Aerial part			

**Table 3:** Use report (UR), use value (UV), frequency of citation (FC), relative frequency of citation (RFC), cultural importance (CI), relative importance (RI), and number of uses for veterinary.

No.	Species name	URs	UV	FCs	RFCs	CI	RIs	NUs
1	*Achillea eriophora* DC.	1	0.005	1	0.005	0.005	0.064	1
2	*Prunus eburnea* (Spach) Aitch.	10	0.05	5	0.025	0.05	0.321	5
3	*Amygdalus scoparia* Spach	1	0.005	1	0.005	0.005	0.064	1
4	*Matricaria aurea* (Loefl.) Sch.Bip.	2	0.01	1	0.005	0.01	0.12	2
5	*Astragalus fasciculifolius* Boiss.	16	0.08	14	0.07	0.08	0.287	3
6	*Berberis* sp.	1	0.005	1	0.005	0.005	0.064	1
7	*Calotropis procera* (Aiton) W.T.Aiton	1	0.005	1	0.005	0.005	0.064	1
8	*Camellia sinensis* (L.)	2	0.01	2	0.01	0.01	0.073	1
9	*Capparis spinosa* L.	6	0.03	5	0.025	0.03	0.21	3
10	*Citrus × aurantiifolia* (Christm.) Swingle	1	0.005	1	0.005	0.005	0.064	1
11	*Coffea arabica* L.	1	0.005	1	0.005	0.005	0.064	1
12	*Crataegus aronia* (L.) Steud.	2	0.01	2	0.01	0.01	0.073	1
13	*Curcuma longa* L.	4	0.02	2	0.01	0.02	0.128	2
14	*Ephedra pachyclada* Boiss.	4	0.02	1	0.005	0.02	0.231	4
15	*Eruca sativa* Miller	2	0.01	2	0.01	0.01	0.073	1
16	*Haplophyllum laristanicum* C.C.Towns.	4	0.02	4	0.02	0.02	0.146	2
17	*Ferula assa-foetida* Boiss.	88	0.44	58	0.29	0.44	1	9
18	*Ficus johannis* Boiss.	1	0.005	1	0.005	0.005	0.064	1
19	*Glycyrrhiza glabra* L.	1	0.005	1	0.005	0.005	0.064	1
20	*Otostegia persica* (Burm.) Boiss.	6	0.03	5	0.025	0.03	0.154	2
21	*Periploca graeca* L.	6	0.03	6	0.03	0.03	0.163	2
22	*Phoenix dactylifera* L.	2	0.01	2	0.01	0.01	0.073	1
23	*Prunus amygdalus* Batsch	1	0.005	1	0.005	0.005	0.064	1
24	*Sesamum indicum* L.	1	0.005	1	0.005	0.005	0.064	1
25	*Tecomella undulata* (Sm.) Seem.	6	0.03	3	0.015	0.03	0.137	2
26	*Teucrium polium* L. (C)	1	0.005	1	0.005	0.005	0.064	1
27	*Trigonella foenum-graecum* L.	4	0.02	2	0.01	0.02	0.184	3
28	*Triticum aestivum* L.	3	0.015	3	0.015	0.015	0.137	2
29	*Zataria multiflora* Boiss.	3	0.015	3	0.015	0.015	0.081	1
30	*Ziziphus nummularia* (Burm.f.) Wight. & Arn.	5	0.025	5	0.025	0.025	0.099	1
31	*Ziziphus spina-christi* (L.) Desf.	5	0.025	5	0.025	0.025	0.099	1

**Table 4:** Informant consensus factor for commonly used medicinal plants.

Ailments	Nur	Ntaxa	ICF
Animal bite	9	5	0.5
Digestive	70	11	0.86
Epistaxis	3	3	0
Fever	1	1	0
Fractures	30	10	0.69
Grass tetany	6	3	0.6
Increasing milk production	4	3	0.33
Infected wound	11	5	0.6
Lambing	3	3	0
Muscle and joint pain	40	9	0.79
Respiratory	4	2	0.67
Scabies	2	1	1
Udder problems	5	3	0.5
Urinary retention	1	1	0
Weaning	1	1	0

**Table 5:** Plants with the highest choice value for livestock diseases.

Ailments	Plants with the highest choice value	CV
Animal bite	*Periploca graeca* L.	1.67
	*Tecomella undulata* (Sm.) Seem.	1
Digestive	*Ferula assa-foetida* Boiss.	4.27
	*Ziziphus* sp.	0.45
Fractures	*Astragalus fasciculifolius* Boiss.	1.3
	*Otostegia persica* (Burm.) Boiss.	0.5
Grass tetany	*Camellia sinensis* (L.) Kuntze	0.67
	*Ferula assa-foetida* Boiss.	0.67
	*Phoenix dactylifera* L.	0.67
Increasing milk production	*Amygdalus lycioides* Spach	0.66
Infected wound	*Capparis spinosa* L.	0.6
	*Ferula assa-foetida* Boiss.	0.6
Muscle and joint pain	*Ferula assa-foetida* Boiss.	3.22
	*A. lycioides* Spach	0.44
Respiratory	*Ferula assa-foetida* Boiss.	1
	*Trigonella foenum-graecum* L.	1
Scabies	*Eruca sativa* Miller	2
Udder problems	*T. undulata* (Sm.) Seem	1
Urinary retention	*Crataegus aronia* (L.) Steud.	2

**Table 6:** Fidelity level values of the species by ailment in veterinary medicine.

Primary use	Species name	FLs
Animal bite	*Tecomella undulata* (Sm.) Seem.	100
Animal bite	*Teucrium polium* L. (C)	100
Animal bite	*Periploca graeca* L.	83.33
Digestive	*Achillea eriophora* DC.	100
Digestive	*Amygdalus lycioides* Spach	40
Digestive	*Coffea arabica* L.	100
Digestive	*Ephedra pachyclada* Boiss.	100
Digestive	*Haplophyllum laristanicum* C.C.Towns.	75
Digestive	*Ferula assa-foetida* Boiss.	81.03
Digestive	*Trigonella foenum-graecum* L.	50
Digestive	*Triticum aestivum* L.	33.33
Digestive	*Zataria multiflora* Boiss.	100
Digestive	*Ziziphus nummularia* (Burm.f.) Wight. & Arn.	100
Digestive	*Ziziphus spina-christi* (L.) Desf.	100
Epistaxis	*A. lycioides* Spach	20
Epistaxis	*E. pachyclada* Boiss.	100
Epistaxis	*Ferula assa-foetida* Boiss.	1.72
Fever	*Citrus × aurantiifolia* (Christm.) Swingle	100
Fractures	*Amygdalus scoparia* Spach	100
Fractures	*Astragalus fasciculifolius* Boiss.	92.86
Fractures	*Capparis spinosa* L.	40
Fractures	*Curcuma longa* L.	100
Fractures	*Ferula assa-foetida* Boiss.	3.45
Fractures	*Glycyrrhiza glabra* L.	100
Fractures	*Otostegia persica* (Burm.) Boiss.	100
Fractures	*Prunus amygdalus* Batsch	100
Fractures	*Triticum aestivum* L.	66.67
Fractures	*P. graeca* L.	16.67
Grass tetany	*Camellia sinensis* (L.) Kuntze	100
Grass tetany	*Ferula assa-foetida* Boiss.	3.45
Grass tetany	*Phoenix dactylifera* L.	100
Increasing milk production	*A. lycioides* Spach	40
Increasing milk production	*E. pachyclada* Boiss.	100
Increasing milk production	*Ferula assa-foetida* Boiss.	1.72
Infected wound	*A. fasciculifolius* Boiss.	14.29
Infected wound	*Calotropis procera* (Aiton) W.T.Aiton	100
Infected wound	*C. spinosa* L.	60
Infected wound	*Curcuma longa* L.	100
Infected wound	*Ferula assa-foetida* Boiss.	5.17
Lambing	*A. lycioides* Spach	20
Lambing	*Matricaria aurea* (Loefl.) Sch.Bip.	100
Lambing	*C. spinosa* L.	20
Muscle and joint pain	*A. lycioides* Spach	80
Muscle and joint pain	*Berberis* sp.	100
Muscle and joint pain	*E. pachyclada* Boiss.	100
Muscle and joint pain	*H. laristanicum* C.C.Towns.	25
Muscle and joint pain	*Ferula assa-foetida* Boiss.	50
Muscle and joint pain	*Ficus johannis* Boiss.	100
Muscle and joint pain	*O. persica* (Burm.) Boiss.	20
Muscle and joint pain	*Sesamum indicum* L.	100
Muscle and joint pain	*Trigonella foenum-graecum* L.	50
Respiratory	*Ferula assa-foetida* Boiss.	3.45
Respiratory	*Trigonella foenum-graecum* L.	100
Scabies	*Eruca sativa* Miller	100
Udder problems	*Matricaria aurea* (Loefl.) Sch.Bip.	100
Udder problems	*Ferula assa-foetida* Boiss.	1.72
Udder problems	*T. undulata* (Sm.) Seem.	100
Urinary retention	*Crataegus aronia* (L.) Steud.	100
Weaning	*A. fasciculifolius* Boiss.	7.14

## Data Availability

Data are available on request from the authors.

## Nomenclature

FC: Frequency citation
FL: Fidelity level
ICF: Informant consensus factor
UR: Use reports
UV: Use value
CV: Choice value
PP: Plant part
AP: Aerial parts
